# Marine Science: Surf’s Yuck

**DOI:** 10.1289/ehp.112-a614b

**Published:** 2004-08

**Authors:** Tina Adler

To get the real skinny on the health effects of coastal water pollution, talk to a surfer. While catching the waves, surfers are also catching colds, stomach bugs, and more. Surfers long ago made the connection between sick days and urban storm drains dumping untreated runoff from streets, yards, and waterways into beach water. But researchers have now calculated the likelihood of surfers succumbing to waterborne bacteria and viruses.

Environmental scientist Ryan H. Dwight of the University of California, Irvine, and colleagues interviewed 1,873 surfers in two California surfing hot spots: rural Santa Cruz County and urban northern Orange County. The researchers interviewed the surfers in April 1998, following a very wet El Niño winter with greater runoff than usual, and again in April 1999, following a very dry La Niña winter with less runoff than usual.

The first year, Orange County surfers reported almost twice as many symptoms over the previous three months compared with Santa Cruz surfers. Their symptoms included fever, nausea, stomach pain, sore throats, and eye, ear, and skin infections, the team reported in the April 2004 *American Journal of Public Health*. But even Santa Cruz surfers weren’t entirely safe that spring. Every additional 2.5 hours that surfers in either county spent in the water increased by 10% their likelihood of developing symptoms, the team writes. In the spring following the drier La Niña winter, Orange County surfers reported only slightly more symptoms than Santa Cruz participants.

All of the participants, whose mean age was 30, surfed at least once a week. For their water quality data, the researchers used mean monthly total coliform counts collected by the two counties’ health agencies. Orange County scored much worse on water quality tests in the first year than did Santa Cruz, which is a small, less-developed watershed.

Since the study was done, California has expanded its water quality testing requirements. In 1999, in accordance with updated state standards, California began measuring for enterococci, bacteria that inhabit the intestine. Dwight and others say that although overall water quality may not have improved, the change did result in many more beach closings, particularly in Orange County.

The work by Dwight and colleagues helps confirm in a tangible way what swimmers and surfers know from experience, says Cheryl McGovern, a program manager with the U.S. Environmental Protection Agency in San Francisco. People need studies to quantify the health risks associated with various recreational waters, especially if they will be paying for pollution cleanup, she says. She would like to see a follow-up study that uses more sophisticated water quality data, including measurements of enterococci.

Dwight notes that surfers are not the only people exposed to the waters in these or other coastal counties—several millions of tourists and local residents swim in these waters every year. “If the surfers are getting sick—and they are young and healthy—then the public is at risk as well,” he says.

## Figures and Tables

**Figure:**
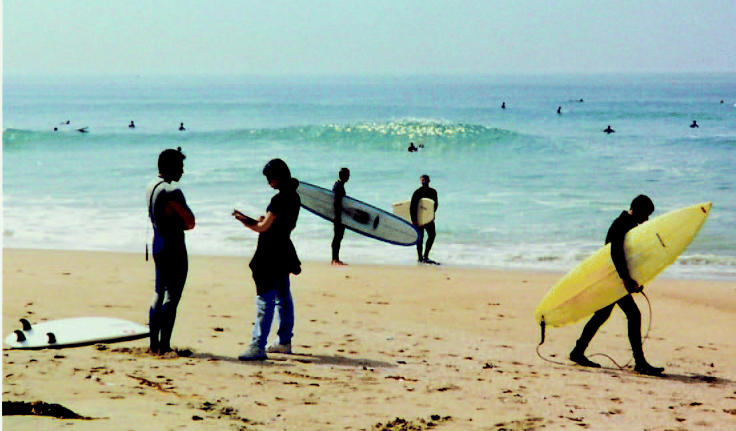
**Dangerous waves.** A study of surfers connects a raft of health symptoms with waters flooded with toxic runoff following storms.

